# Virulence Factors of *Escherichia coli* Isolated From Female Reproductive Tract Infections and Neonatal Sepsis

**DOI:** 10.1155/S1064744901000333

**Published:** 2001

**Authors:** Susan W. Cook, Hunter A. Hammill, Richard A. Hull

**Affiliations:** 1 Department of Biology Houston Baptist University Houston TX USA; 2 Departments of Pediatrics and Family and Community Medicine Baylor College of Medicine Houston TX USA; 3 The Center for Prostheses Infections Baylor College of Medicine Houston TX USA; 4 Departments of Molecular Virology and Microbiology and Physical Medicine and Rehabilitation Baylor College of Medicine One Baylor Plaza Houston TX 77030 USA

## Abstract

*Objective:* The presence of enterobacteria such as *Escherichia coli* in the vagina of normal women is not synonymous with infection. However, vaginal *E. coli* may also cause symptomatic infections. We examined bacterial virulenceproperties that may promote symptomatic female reproductive tract infections (RTI) and neonatal sepsis.

*Methods:* * E. coli * isolated as the causative agent from cases of vaginitis (n = 50), tubo-ovarian abscess (n = 45) and neonatal sepsis (n = 45) was examined for selected phenotypic and genetic virulence properties. Results were compared with the frequency of the same properties among fecal *E. coli* not associated with disease.

*Results:* A significantly greater proportion of infection *E. coli* exhibited D-mannose resistant hemagglutination compared with fecal *E. coli* (*p* < 0.01). This adherence phenotype was associated with the presence of P fimbriae (*pap*) genes which were also significantly more prevalent among isolates from all three infection sites (*p* < 0.01). The majority of *pap*^+^ isolates contained the *papG3* allele (Class II) regardless of infection type. Increased frequency of Type 1C genes among vaginitis and abscess isolates was also noted. No significant differences in frequency of other bacterial adherence genes, fim, sfa, uca (gaf) or dra were observed. E. coli associated with vaginitis was significantly more likely to be hemolytic ( HIy^+^) than were fecal isolates (*p* < 0.05). The HIy^+^ phenotype was also more prevalent among tubo-ovarian abscess and neonatal sepsis isolates (*p* < 0.08).

*Conclusions:* *E. coli* isolated from female RTI and neonatal sepses possess unique properties that may enhance their virulence. These properties are similar to those associated with other *E. coli* extra-intestinal infections, indicating that strategies such as vaccination or bacterial interference that may be developed against urinary tract infections (UTI) and other *E. coli* extra-intestinal infections may also prevent selected female RTI.


*Infect Dis Obstet Gynecol 2001;9:203–207*

### Virulence factors of *Escherichia coli* isolated from female reproductive tract infections and neonatal sepsis

**Susan W. Cook[1], Hunter A. Hammill[2,3] and Richard A. Hull[3,4]**

[1]*Department of Biology, Houston Baptist University, Houston, TX*
[2]*Departments of Pediatrics and Family and Community Medicine, Baylor College of Medicine, Houston, TX*
[3]*The Center for Prostheses Infections, Baylor College of Medicine, Houston, TX*
[4]*Departments of Molecular Virology and Microbiology and Physical Medicine and Rehabilitation, Baylor College of Medicine, Houston, TX*

***Objective:*** The presence of enterobacteria such as *Escherichia coli* in the vagina of normal women is not synonymous with infection. However, vaginal *E. coli* may also cause symptomatic infections. We examined bacterial virulence properties that may promote symptomatic female reproductive tract infections (RTI) and neonatal sepsis.
***Methods:*** *E. coli* isolated as the causative agent from cases of vaginitis (n = 50), tubo-ovarian abscess (n = 45) and neonatal sepsis (n = 45) was examined for selected phenotypic and genetic virulence properties. Results were compared with the frequency of the same properties among fecal *E. coli* not associated with disease.
***Results:*** A significantly greater proportion of infection *E. coli* exhibited D-mannose resistant hemagglutination compared with fecal *E. coli* ($p < 0.01$). This adherence phenotype was associated with the presence of P fimbriae (*pap*) genes which were also significantly more prevalent among isolates from all three infection sites ($p < 0.01$). The majority of $pap^+$ isolates contained the *papG3* allele (Class II) regardless of infection type. Increased frequency of Type 1C genes among vaginitis and abscess isolates was also noted. No significant differences in frequency of other bacterial adherence genes, *fim*, *sfa*, *uca* (*gaf*) or *dra* were observed.
*E. coli* associated with vaginitis was significantly more likely to be hemolytic ($Hly^+$) than were fecal isolates ($p < 0.05$). The $Hly^+$ phenotype was also more prevalent among tubo-ovarian abscess and neonatal sepsis isolates ($p < 0.08$).
***Conclusions:*** *E. coli* isolated from female RTI and neonatal sepses possess unique properties that may enhance their virulence. These properties are similar to those associated with other *E. coli* extra-intestinal infections, indicating that strategies such as vaccination or bacterial interference that may be developed against urinary tract infections (UTI) and other *E. coli* extra-intestinal infections may also prevent selected female RTI.

Key words: VAGINITIS, TUBO-OVARIAN ABSCESS, UROGENITAL TRACT INFECTION (UTI)

The composition of bacterial flora in the vagina is complex and changes with a multitude of events in the patient's life. While the predominant aerobic flora consist of *Lactobacillus* and *Streptococcus* species, the presence in the vagina of other bacteria, such as *Escherichia coli*, is not synonymous with infection. Indeed the incidence of *E. coli* in the vagina of normal, pre-menopausal, non-pregnant,

Funding: USPHS grant #HD35856
Correspondence to: Richard Hull, Department of Virology and Microbiology, Baylor College of Medicine, One Baylor Plaza, Houston, TX 77030. Email: rhull@bcm.tmc.edu asymptomatic women is about 21%[1]. However, vaginal *E. coli* may also cause symptomatic infections such as vaginitis or tubo-ovarian abscess and is associated with life-threatening neonatal sepsis[2]. Neonates are presumably exposed to *E. coli* during passage through the birth canal[3,4].

*E. coli* isolated from infections possesses unique properties that allow it to colonize and persist at infection sites. These properties are common to many pathogenic *E. coli*, regardless of the site of infection. For example, properties such as high-affinity iron scavenging serve metabolic functions that allow the bacteria to grow. Expression of surface carbohydrate antigens, O and K, protect the bacteria from host immune mechanisms[5]. Other properties, such as adherence fimbriae, are unique to certain pathogenic *E. coli*. For example, P type fimbriae promote specific attachment to kidney tissue and are highly correlated with pyelonephritis, whereas Type 1 fimbriae facilitate attachment to bladder walls and correlate with cystitis[5].

Little is known about virulence properties of *E. coli* that may promote bacterial colonization and cause symptomatic infection in the vagina. In the following study, we examined *E. coli* strains isolated as the causative agent in female genital tract infections (GTI) and neonatal sepsis for their virulence-associated adherence characteristics. The incidence of hemolysin production, a property that is associated with symptomatic bladder infection, was also examined[5].

### SUBJECTS AND METHODS

### Specimen collection

Clinical *E. coli* samples were collected at the Family Practice Clinic, Baylor College of Medicine, Houston, TX. Vaginitis-associated isolates represented the predominant vaginal flora present concurrent with symptoms. Pelvic inflammatory disease (PID)-associated isolates were abdominal specimens from abscesses. Neonatal sepsis isolates were collected from blood cultures. Criteria used for diagnosis of vaginitis and PID were in accordance with current recommendations[6,7]. Fecal isolates were collected from healthy young adults.

### Colony blot hybridization

Bacteria were inoculated as a patch in a grid of 50 squares on duplicate 100 × 15 mm McConkey agar plates. After overnight incubation at 37°C, bacteria from one plate were transferred to a Whatman 541 filter paper by laying the paper on the agar plate and gently pressing with a glass spreader. The filters were then placed bacteria side up on three layers of Whatman #3 filter paper saturated with 0.5 M NaOH, in a 100 × 15 mm glass Petri dish cover, and steamed for 4 min over a beaker of boiling water. After this the filters were immersed in 250 ml of 1 M Tris, 2 M NaCl, pH 7 for 4 min and air-dried.

Hybridizations were performed in 8 inch square heat-sealable plastic bags. Filters were enclosed, two per bag, washed once briefly with 20 ml hybridization buffer (1 × SSC, 1% SDS, 0.5% powdered milk), and then suspended in 20 ml hybridization buffer. Radiolabeled probe DNA (1–2 × $10^6$ DPM) was added to 0.5 ml TE (10 mM Tris, 1 mM EDTA, pH 8) containing 2 mg sheared calf thymus DNA, and denatured in boiling water for 5 min. Denatured probe was added to the bags, which were sealed and incubated overnight at 65°C. After incubation, filters were removed from the bags, washed once briefly at room temperature in 500 ml wash buffer (1 × SSC, 0.1% SDS) and then incubated in wash buffer for 1 h at 68°C. Filters were then washed briefly at room temperature in 1 × SSC, dried and exposed to x-ray film overnight at −70°C. DNA probes used for the detection of adherence gene clusters *fim, dra, pap, sfa*, *foc* and *uca* have been described previously[8–12]. Probes were radiolabeled with $^{32}$P deoxycytidine triphosphate (dCTP) using a random priming kit (Pharmacia Upjohn, Sweden).

### Hemagglutination assays

Bacteria were grown in patches on L agar plates overnight at 37°C. A small amount of bacteria was suspended with a sterile toothpick into a drop of 5% (vol/vol) fresh human erythrocytes suspended in buffered saline (per liter: 8.5 g NaCl, 0.3 g $KH_2PO_4$, 1.2 g $Na_2HPO_4$ $7H_2O$ [pH 7]) containing 50 mM D-mannose. After the bacteria were thoroughly suspended on a glass plate, the plate was gently rocked on ice and the erythrocytes were observed for agglutination.

### Assay of *pap* alleles

Alleles of the *papG* gene that encode either Class I (*papG1*)[13], Class II (*papG3*)[13] or Class III (*papG2*)[14] pili were distinguished using the polymerase chain reaction (PCR) method described by Johnson[15], except that the following thermal cycling program was used: 1) 94°C for 1 min; 2) 60°C for 2 min; 3) 72°C for 3 min; 4) repeat steps 1–3 26 times; and 5) 72°C for 20 min.

### RESULTS

### Adherence and hemolysin phenotypes of infection *E. coli*

A total of 50 *E. coli* isolates from cases of vaginitis, 45 from cases of tubo-ovarian abscess and 45 from cases of neonatal sepsis were collected. Each was grown *in vitro* and tested for ability to hemagglutinate human erythrocytes in the presence of D-mannose (mannose resistant hemagglutination, MRHA). Results were compared with the MRHA phenotype of 50 *E. coli* strains collected from the stools of healthy women. A significantly greater proportion of *E. coli* isolated from infections expressed the MRHA$^+$ phenotype ($p < 0.01$) compared with fecal isolates (Table 1). In addition, when tested for hemolysin production on sheep blood agar plates, *E. coli* associated with vaginitis was significantly more likely to be hemolytic (Hly$^+$) than were fecal isolates ($p < 0.05$). The Hly$^+$ phenotype was also more prevalent among tubo-ovarian abscess and neonatal sepsis isolates than fecal isolates ($p < 0.08$). *E. coli* may also produce a hemagglutinin, called a Type 1 fimbria, that recognizes a mannose-containing tissue receptor. Hemagglutination caused by Type 1 fimbriae is inhibited by the presence of free mannose. Expression of Type 1 pili was not assayed because expression of this pili type *in vitro* does not accurately reflect expression *in vivo* at the infection site[16].

### Frequency of adherence genes

*E. coli* isolated from each infection site was screened using DNA-DNA hybridization for presence of bacterial adherence genes. The genotypes selected for analysis were those considered to be associated with other extra-intestinal infections such as adult sepsis and UTI. They included genes for Type 1 (*fim*), S (*sfa*), P (*pap*), Uca (*uca*, *gaf*), Dr (*dra*) and Type 1C (*foc*) fimbriae. Results in Table 1 show that *pap* genes were significantly more prevalent among isolates from all three infection sites ($p < 0.01$). Increased frequency of Type 1C genes among vaginitis and abscess isolates was also noted. No significant differences in frequency of *fim*, *sfa*, *uca* (*gaf*) or *dra* genes in infection isolates compared with fecal isolates were observed.

Strains that contained *pap* genes were also tested to determine the specific *papG* gene allele present. The results are shown in Table 2. The majority of *pap* isolates contained the *papG3* allele (Class II) solely or in combination with the *papG2* allele (Class III), regardless of infection type.

### DISCUSSION

Previous studies have shown that *E. coli* isolated from extra-intestinal infections possesses unique

**Table 1** Virulence factors of female reproductive tract infection isolates

| | *Number (%) of isolates with tested phenotype* | | *Number (%) of isolates with tested genotype* | | | | | |
|---|---|---|---|---|---|---|---|---|
| *Source* | *MRHA* | *HLY* | pap | pil | foc | sfa | uca | dra |
| Vaginitis (n = 50) | 24 (48)* | 14 (28)** | 23 (46)* | 45 (90) | 9 (18)** | 7 (14) | 5 (10) | 7 (14) |
| Tubo-ovarian abscess (n = 45) | 22 (49)* | 11 (24) | 18 (41)* | 38 (86) | 6 (13)** | 5 (11) | 2 (5) | 8 (18) |
| Neonatal sepsis (n = 45) | 25 (56)* | 11 (24) | 18 (40)* | 41 (90) | 5 (11) | 4 (9) | 2 (5) | 8 (18) |
| Fecal (n = 50) | 8 (16) | 6 (12) | 5 (10) | 44 (87) | (4)† | 8 (16) | 3 (6) | 8 (16) |

*$p < 0.01$ compared with fecal; **$p < 0.05$ compared with fecal; †data obtained from Mitsumori *et al.*[11]; MRHA, D-mannose resistant hemagglutination of human or sheep erythrocytes; HLY , expression of hemolysin

**Table 2** Frequency of specific *papG* alleles among infection isolates

| *Source* | *Class I* (papG1) | *Class II* (papG3) | *Class III* (papG2) | *Class II + III* | *Other** |
|---|---|---|---|---|---|
| Vaginitis (n = 20) | 1 (5%) | 15 (75%) | 2 (10%) | 2 (10%) | 0 |
| Tubo-ovarian abscess (n = 17) | 0 | 13 (76%) | 1 (6%) | 1 (6%) | 1 |
| Neonatal sepsis (n = 18) | 0 | 16 (89%) | 0 | 0 | 2 |

*Produced PCR product with a size different from the three previously identified *papG* alleles, or no product properties that distinguish it from normal fecal flora *E. coli*[10]. We have extended these studies to show that *E. coli* isolated from female reproductive tract infections (RTI) also possesses specific properties that may promote its virulence. Bacterial adherence to mucosal surfaces is a key step in the colonization/infection process. Nearly half of the *E. coli* strains representing all infection sites examined exhibited an MRHA-positive phenotype. This *in vitro* phenotype is a proxy for specific adherence to epithelial tissue and has been linked to bacterial virulence[5]. For example, the MRHA phenotype has been demonstrated to be strongly associated with kidney infection and urosepsis.

MRHA is a consequence of expression of one or more types of fimbrial adhesins on the bacterial cell surface. Fimbriae are sorted into groups based upon their tissue receptor specificity. For example, Dr fimbriae, encoded by *dra* genes, recognize and bind to the Dr peptide antigen present on human tissue, whereas P fimbriae bind to the P (or the structurally related Luke) carbohydrate antigen[5,14]. Different types of fimbriae may be detected and distinguished from each other based upon the unique genotype that encodes their expression. Genes associated with one or more D-mannose resistant fimbriae types were detected in 60% of vaginitis and neonatal sepsis isolates and in 70% of abscess isolates. P fimbriae genes were the predominant type present. P fimbriae may contribute to symptomatic RTI just as they do in about 80% of *E. coli* isolated from pyelonephritis infections: in UTI, P fimbriae mediate specific attachment of uropathogenic *E. coli* to kidney tissue and elicit a cytokine response in those cells[5,17]. The role of P fimbriae in genital tract infections (GTI) is not currently known.

There are at least three classes of P fimbriae, based on genetic diversity and on attachment to different – but related – tissue receptors. Previous studies of *E. coli* isolated from UTI and adult urosepsis revealed that Class II P fimbriae are the most prevalent class[5,18]. Stapleton and colleagues[19] also reported that Class II and/or Class III P fimbriae were most frequent among P fimbriated *E. coli* isolated as asymptomatic colonizers of the vagina. However, this study did not distinguish between Class II and Class III P fimbriae. Results presented in the current study show that Class II P fimbriae are significantly more prevalent than other P fimbriae classes on *E. coli* isolated from symptomatic infections. Overall, these results suggest that P fimbriae, and possibly Type 1C fimbriae, contribute to *E. coli* GTI.

The possible role of Type 1 fimbriae in the colonization/infection process was not addressed in this study. The use of a clinical correlation approach is less helpful for determining a possible role for Type 1 fimbriae. Comparison of the frequency of *fim* genes between bacteria isolated from infections and avirulent bacteria confirmed other studies showing that *fim* genes are present in nearly all *E. coli* isolates. Also, unlike for other fimbrial types, Type 1 fimbriae may be expressed at the site of infection but not expressed under laboratory conditions. For example, Hultgren and colleagues[16] have used a murine cystitis UTI model to study expression of Type 1 fimbriae. They found that while bacteria that were attached to the bladder walls expressed fimbriae, bacteria that were collected from the lumen or that were subcultured *in vitro* did not express fimbriae. As a consequence, analysis of Type 1 fimbrial expression *in vitro*, such as by measuring hemagglutination, is not revealing. Type 1 fimbriae contribute significantly to colonization of the bladder[20,21] and may contribute to reproductive tract colonization as well. Future studies will examine the role of type 1 fimbriae in promoting infections.

In conclusion, *E. coli* isolated from female RTI and neonatal sepsis possess unique properties that may enhance their virulence. These properties are similar to those associated with other *E. coli* extra-intestinal infections, indicating that strategies such as vaccination[20] or bacterial interference[21] that may be developed against UTI and other *E. coli* extra-intestinal infections, may also be effective for prevention of selected female RTI.

### REFERENCES

1. Hammill H. Normal vaginal flora in relation to vaginitis. *Obstet Gynecol Infect* 1989;16:329–36
2. Percival-Smith R. Vaginal colonization of Escherichia coli and its relation to contraceptive methods. *Contraception* 1983;27:497–504
3. Crombleholme WR, Ohm-Smith M, Robbie MO, *et al.* Ampicillin/sulbactam versus metronidazole-gentamicin in the treatment of soft tissue pelvic infections. *Am J Obstet Gynecol* 1987; 156(2):507–12
4. Krohn MA, Thwin SS, Rabe LK, *et al.* Vaginal colonization by *Escherichia coli* as a risk factor for very low birth weight delivery and other perinatal complications. *J Infect Dis* 1997;175:606–10
5. Johnson JR. Virulence factors in *Escherichia coli* urinary tract infection. *Clin Micobiol Rev* 1991;4: 80–128
6. French JI, McGregor JA. Bacterial vaginosis. In Faro S, Soper DE, eds. *Infectious Diseases in Women*. Philadelphia: Saunders, 2001:221–39
7. Soper DE. Pelvic Inflammatory Disease. In Faro S, Soper DE, eds. *Infectious Diseases in Women*. Philadelphia: Saunders, 2001:267–78
8. Buchanan K, Falkow S, Hull RA, *et al.* Frequency among *Enterobacteriaceae* of the DNA sequences encoding Type 1 pili. *J Bacteriol* 1985;162:799–803
9. Nowicki BJ, Svanborg-Eden C, Hull R, *et al.* Molecular analysis and epidemiology of the DR hemagglutinin of uropathogenic *Escherichia coli*. *Infect Immun* 1989;57:446–51
10. Hull RA, Hull SI, Falkow S. Frequency of gene sequences necessary for pyelonephritis associated pili expression among isolates of *Enterobacteriaceae* from human extraintestinal infections. *Infect Immun* 1984;43:1064–7
11. Mitsumori K, Terai A, Yamamoto S, *et al.* Identification of S, F1C, and three PapG fimbrial adhesins in uropathogenic *Escherichia coli* by polymerase chain reaction. *FEMS Immunol Med Microbiol* 1998;21:261–8
12. Hull RA, Rudy DC, Donovan WH, *et al.* Virulence properties of *E. coli* 83972, a prototype strain associated with asymptomatic bacteriuria. *Infect Immun* 1999;67:429–32
13. Klann AG, Hull RA, Hull SI. Sequences of the genes encoding the minor tip components of Pap-3 pili of *Escherichia coli*. *Gene* 1992;119:95–100
14. Karr JF, Nowicki BJ, Troung LD, *et al.* *pap-2* encoded fimbriae adhere to the P blood group-related glycosphingolipid stage-specific embryonic antigen 4 in the human kidney. *Infect Immun* 1990; 58:4055–62
15. Johnson, JR. *papG* alleles among *Escherichia coli* strains causing urosepsis: associations with other bacterial characteristics and host compromise. *Infect Immun* 1998;66:4568–71
16. Hultgren SJ, Porter TN, Schaeffer AJ, *et al.* Role of Type 1 pili and effects of phase variation on lower urinary tract infections produced by *Escherichia coli*. *Infect Immun* 1985;50:370–7
17. Hedlund M, Svensson M, Nilsson A, *et al.* Role of the ceramide pathway in cytokine responses to P-fimbriated *Escherichia coli*. *J Exp Med* 1996;183: 1037–44
18. Johnson JR, Russo TA, Brown JJ, *et al.* *papG* alleles of *Escherichia coli* strains causing first episode or recurrent acute cystitis in adult women. *J Infect Dis* 1998;177:97–101
19. Stapleton A, Hooten TM, Fennell C, *et al.* Effect of secretor status on vaginal and rectal colonization with fimbriated *Escherichia coli* in women with and without recurrent urinary tract infection. *J Infect Dis* 1995;171:717–20
20. Langermann S, Palaszynski S, Branhart M, *et al.* Prevention of mucosal *Escherichia coli* infection by FimH-adhesin-based systemic vaccination. *Science* 1997;276:607–11
21. Hull RA, Rudy DC, Donovan WH, *et al.* Clinical outcome of intentional bladder colonization with *E. coli* 83972. *J Urol* 2000;163:872–7

Received 05/23/01; Accepted 09/27/01
